# The efficacy and safety of epidural morphine/hydromorphone in the treatment of intractable postherpetic neuralgia: A single-center, double-blinded, randomized controlled, prospective, and non-inferiority study

**DOI:** 10.3389/fphar.2022.1051357

**Published:** 2022-12-06

**Authors:** Sun Yiping, Shen Jiayi, Hei Guang, Ji Yun, Ma Bingjie, Huang Xuehua, Yu Zhiyuan, Ma Pingchuan, Ma Ke

**Affiliations:** ^1^ Department of Clinical Research, Xinhua Hospital, Medical School, Shanghai Jiaotong University, Shanghai, China; ^2^ Department of Algology, The Second Affiliated Hospital of Zhengzhou University, Zhengzhou, China; ^3^ Department of Algology, Xinhua Hospital, Medical School, Shanghai Jiaotong University, Shanghai, China; ^4^ High School of Jiguang, Hongkou District, Shanghai, China

**Keywords:** PHN (postherpetic neuralgia), epidural, morphine, hydromorphone, VAS (visual analogue scale)

## Abstract

**Objective:** Postherpetic neuralgia (PHN) is a clinical puzzle, especially in patients who still suffered from moderate and severe pain after standard treatment. This single-center, double-blinded, randomized controlled, prospective, and non-inferiority study observed the safety and effectiveness of the epidural application of morphine or hydromorphone, trying to provide an alternative method for those patients with refractory PHN.

**Methods:** Eighty PHN patients with a visual analogue scale (VAS) still greater than 50 mm after routine management were randomly divided into two groups according to 1:1, respectively. One group received epidural morphine (EMO group), and the other group received epidural hydromorphone (EHM group). VAS, the number of breakthrough pain, quality of life (QOL), and anxiety/depression assessment (GAD-7 and PHQ-9 scores) were also observed before treatment, at 1, 3, 7, 14, 21, 28, 60, and 90 days after treatment, as well as side effects. Opioid withdrawal symptoms (OWSs) were also measured from 3 to 28 days after treatment.

**Results:** The EHM group was non-inferior to the EMO group in terms of the VAS decrease relative to baseline (VDRB) after 1-week treatment. The VAS of the two groups on all days after treatment was significantly lower than the corresponding baseline findings (*p* < 0.05). The breakthrough pain (BTP) decreased significantly after treatment and lasted until 14 days after treatment (*p* < 0.05). There was no significant difference in BTP between the two groups at each time point (*p* > 0.05). In terms of the QOL, GAD-7, and PHQ-9 outcomes, those were significantly improved after treatment (*p* < 0.05), and there was no difference between the two groups (*p* > 0.05). No significant AE difference across the two groups was observed in this study. Few reports of OWS were found in this trial, and there were no significant differences between the two groups (*p* > 0.05).

**Conclusion:** EHM was non-inferior to EMO in terms of the VDRB after 1-week treatment. For patients with VAS still greater than 50 mm after standard treatment, short-term application of EMO or EHM can ameliorate intractable pain, improve the quality of life, and have no obvious side effects. Short-term epidural opioid application will not lead to the appearance of OWS.

## 1 Introduction

PHN is known as one of the clinical challenges not only in pain departments but also in dermatology and neurology patients. Moreover, the incidence of herpes zoster and postherpetic neuralgia has increased in recent years, which has a serious negative impact on the patient’s quality of life (Ke et al., 2013; Johnson and Rice, 2014). Some acute herpes zoster patients recover with the treatment protocol recommended by IASP and CASP. There are still some patients who could not get adequate pain relief and eventually develop postherpetic neuralgia, the disease course of which could last as long as 10 years. For patients whose pain is difficult to control with various treatments, most of it is closely related to the patients’ older age, the number of herpes, severe immune response caused by herpes zoster virus, and the severity of neurological damage (Jung et al., 2004; Yang et al., 2019). For patients with refractory PHN, many would take oral pregabalin up to 600 mg/day. On top of that, tricyclic antidepressants, selected serotonin/norepinephrine reuptake inhibitors, and a topical lidocaine transdermal patch are also used for pain control (Hempenstall et al., 2005; Gross et al., 2020). Some patients even received paravertebral nerve blocks for the corresponding dermatome when not achieving satisfactory pain control. The patients’ mood, emotional state, sleep state, and overall quality of life are all seriously affected as a consequence. With this background, providing patients with effective clinical treatment strategies and achieving immediate pain relief are particularly important. The use of opioids can effectively control all kinds of refractory pain, but there are no clinical data on which opioids to be used, the route of administration, and whether there are adverse reactions to opioid withdrawal. There is not enough reference or basis for opioid clinical application. With these considerations, we aim to explore the safety and efficacy of epidural opioid use in refractory PHN treatment as well as the occurrence of opioid withdrawal syndrome. Meanwhile, EMO is a common method, so is EHM; there is no comparative report on this treatment scheme. Therefore, we want to make a comparative evaluation of the effectiveness and safety of these two treatment schemes through this trial. We carried out a single-center, randomized controlled, double-blinded, prospective, and non-inferiority clinical study.

## 2 Materials and methods

### 2.1 Clinical trial design

This is a single-center, prospective, double-blinded, randomized controlled, and non-inferiority clinical study to document the clinical effectiveness and safety of epidural morphine therapy compared with epidural HM in subjects with chronic, intractable, neuropathic pain due to PHN. The study was conducted from 10/03/2019 to 30/05/2020. The study was conducted in accordance with the Good Clinical Practices as outlined in the US Code of Federal Regulations and the Declaration of Helsinki (version 2013). The study protocol was approved by the Human Ethics Committee of Shanghai Xinhua Hospital (Ethics ID: XHEC-C-2019-015-1). This trial was registered with controlled-trials.com (clinical study registration number: ISRCTN17538725).

### 2.2 Patient profile and randomization

Informed consent was signed by patients before being recruited into the study. The clinical research started after eligible patients were admitted to a pain ward. We adopted a computer-generated random assignment sequence. Eighty PHN patients were randomized 1:1 into two groups: the EMO group and the EHM group. The epidural drug in the EMO group (40 cases) was morphine hydrochloride injection (produced by Liaoning Northeast Pharmaceutical Co., Ltd., China). The epidural drug in the EHM group was hydromorphone injection (produced by Hubei Yichang Renfu Pharmaceutical Co., Ltd.). All patient data were included: gender, age, weight, duration of PHN, BMI, VAS, and DN4. The details of baseline characteristics and analgesic therapy of the patients are listed in Table 1.

**TABLE 1:** Baseline characteristics and analgesic therapy of the patients (n = 80). From the summary of demographic data, the average age of the two groups was around 70 years old, and the pain time of PHN was about 3 months on average. The pain level in both groups was moderate to severe, and the average number was 64.6–68.1 mm, and an average of DN4 was 4.98–5.13, all of which were typical neuropathic pain. Among them, the average BMI of the EMO group was 23.2, and the average of the EHM group was 24.7, it was statistically significant difference (*p* = 0.045). Other basic information such as age, gender, weight, pain time, VAS in the two groups, and DN4, etc. were not significantly different.

	EMO (N=40)	EHM (N=40)	*p*-value
Sex			
Female	18 (45.0%)	22 (55.0%)	0.502
Male	22 (55.0%)	18 (45.0%)	
Age			
Mean (SD)	72.6 (6.63)	70.1 (6.90)	0.103
Median (min, max)	72.0 (61.4, 89.0)	68.0 (61.7, 88.6)	
Weight			
Mean (SD)	69.4 (7.04)	68.2 (7.25)	0.45
Median (min, max)	70.4 (50.5, 81.0)	69.2 (52.8, 80.3)	
Pain duration			
Mean (SD)	3.34 (5.42)	3.20 (5.88)	0.911
Median (min, max)	2.00 (1.00, 36.0)	2.10 (1.10, 39.0)	
BMI			
Mean (SD)	23.2 (3.42)	24.7 (3.00)	0.0451
Median (min, max)	22.9 (17.9, 29.2)	25.0 (19.3, 30.2)	
VAS			
Mean (SD)	64.6 (9.39)	68.1 (10.6)	0.131
Median (min, max)	65.0 (51.0, 84.0)	66.5 (45.0, 87.0)	
DNA			
Mean (SD)	5.13 (0.853)	4.98 (0.862)	0.436
Median (min, max)	5.00 (4.00, 7.00)	5.00 (4.00, 7.00)	

### 2.3 Inclusion criteria

The inclusion criteria were as follows:
1. 50 years ≤ age ≤80 years, regardless of gender.
2. Rash onset ≥30 days and VAS≥ 50 mm.
3. Standard neuropathic pain treatment has been performed according to relevant guidelines (IASP, CASP, etc.).
4. Ability to objectively describe symptoms, actively follow doctor’s medication recommendations, and cooperate with the doctor in diagnosis, treatment, and follow-up.
5. Willing to sign written informed consent for study participation.

### 2.4 Exclusion criteria

The exclusion criteria were as follows:
1. Contraindications for epidural catheters or opioids.
2. Allergies to trial-related drugs or devices.
3. Cognitive deficits affecting the ability to assess pain or relieve pain.
4. Have used hydromorphone or morphine during shingles treatment.
5. Pregnant or plan to become pregnant during the study.
6. History of alcohol or drug use.
7. Severe liver and/or kidney damage.
8. History of epidural anesthesia in the past 3 months.
9. Be concomitantly participating in another clinical study.

### 2.5 Provision of medicines and double-blinded

The epidural opioid injections used by the patient are as follows: morphine hydrochloride injection (Northeast Pharmaceutical Co., Ltd., Liaoning, China) and hydromorphone hydrochloride injection (Hubei Yichang Renfu Pharmaceutical Co., Ltd.). All of the aforementioned drugs have been approved in China for clinical pain management. These drugs were applied by clinicians under the principles of government and hospital regulations. Based on a random assignment, an unblinded staff/nurse at the pharmacy prepared the medication before surgery in accordance with GMP. Doctors, follow-up staff, and patients were unaware of the drugs used, and professional emergency unblinding was assigned.

### 2.6 Treatment program

According to clinical manifestations such as pain area and rash distribution, pigmentation, and hyperalgesia in patients with PHN, the nerve segments involved in PHN were identified. The patient’s routine examinations were normal, and there were no contraindications for epidural puncture. The patient entered the DSA operating room of the pain department and underwent ECG, blood pressure, SPO_2_, and respiration monitoring lying in the prone position. After confirming that there was no special situation, routine disinfection, draping of towels, and percutaneous puncture were performed. Under the guidance of fluoroscopy, the epidural puncture is usually performed at 2–3 stages below the segment of the involved nerve, and the epidural catheter is gently placed in the epidural space. The catheter tip is placed at the spinal segment corresponding to the peripheral afferent sensory nerves of the affected skin. The extracorporeal portion of the catheter is then temporarily secured and connected to an external electronic infusion pump with PCA infusion capability. A pre-prepared reservoir bag containing morphine solution (5 mg/100 ml NS) or hydromorphone solution (1 mg/100 ml NS) in the blind state is installed to the pump. The initial infusion rate was 1 ml/h. The patient-controlled analgesia infusion rate was set at 1 ml/press/6 h (Bedder, 1996).

The continuous infusion time was 72 h, calculated from the start time, and the infusion was stopped after 72 h. Immediately after the infusion, the epidural catheter was removed, and the total patient participation time from the admission screening to the end of the treatment period was approximately 4–5 days. All patients’ previous PHN treatment strategies were unaffected throughout the treatment.

### 2.7 Outcome measures

#### 2.7.1 Primary outcome measures

The main non-inferiority evaluation index was the decrease in VAS relative to baseline after 1 week.

#### 2.7.2 Secondary outcome measures

##### 2.7.2.1 Visual analogue scale

Twice a day, the average of daily VAS was calculated at 09:00 and 21:00. Pain assessment time points were baseline, day 1, day 3, day 7, day 14, day 21, day 28, day 60, and day 90 before and after treatment.

##### 2.7.2.2 Breakthrough pain

Pain breakthrough/day was measured using pain diaries recorded by participants. The recording time points were baseline, day 1, day 3, day 7, day 14, day 21, day 28, day 60, and day 90 after treatment.

##### 2.7.2.3 Quality of life, GAD-7, and PHQ-9

Quality of life (QOL) and anxiety/depressive disorders (scored with GAD-7 and PHQ-9) were assessed at baseline, days 7, 14, 28, 60, and 90 after treatment.

QOL is measured by the EURO QOL. We would like to know how good or bad the patient’s health is “today.” This line is numbered from 0 to 100. 100 represents the best health one can imagine. 0 represents the worst health one can imagine (https://euroqol.org).

The GAD-7 scale and the PHQ-9 scale were used to evaluate the patient’s anxiety/depression state. The results of the two scales were scored. The higher the score the more severe the anxiety and depression were.

##### 2.7.2.4 The incidence of adverse reactions and the incidence of opioid withdrawal symptoms

Documentation of safety and tolerability includes opioid-related adverse effects and epidural-related complications. The incidence of adverse reactions and opioid withdrawal symptoms was measured throughout the study using patient self-reports, review of patient notes, patient interviews, medical records, etc. The severity was recorded and decided whether it needs to be dealt with or not. Adverse reactions mainly recorded were nausea, vomiting, urinary retention and itching, and epidural puncture site infection. OWSs primarily recorded were the incidence of palpitations, lacrimation, sweating, and yawning.

##### 2.7.2.5 The correlation plot of breakthrough pain, quality of life, PHQ-9, and GAD-7 versus visual analogue scale of the treatment group.

The circle, triangle, square, and inverse triangle represent the average percent change of BTP, QOL, PHQ-9, and GAD-7, respectively, from baseline at each VAS bin (grouped by 5 mm). The peacock blue and orange colors represent the EMO and EHM groups, respectively.

#### 2.7.3 Statistical analysis

##### 2.7.3.1 Sample size

The sample size was calculated based on the VAS decrease relative to baseline after 7 days of treatment in patients with PHN. If a one-sided alpha level of 2.5% was applied, assuming an SD of 10 for both groups, then 34 individuals per group would be required to achieve a power of 90% for a non-inferiority margin of 8 mm VAS changes. The dropout rate without prolapse recurrence was expected to be about 15%. Thus, a total of 80 participants (40 per group) were determined to be required.

### 2.8 Statistical analysis

The primary non-inferiority measure was the VDRB after 1-week treatment. The evaluation was performed using a one-sided alpha level at 2.5% and a non-inferiority margin of 8 mm VAS. A decline in VAS was continuously measured for 90 days as a secondary indicator. Other secondary measures included 90-day breakout pain, QOL, GAD-7, and PHQ-9. The *t*-test was used to analyze the differences between the two groups at different times and the relative baseline changes in each group. If the data are normally distributed, the repeated measures analysis of variance will also be used to evaluate the differences within each group and time; otherwise, the Wilcoxon signed-rank test will be carried out instead of the *t*-test. For AE, Fisher’s precision probability test was used to compare differences between groups.

Statistical analysis was performed using SAS (version 9.2, SAS Institute, Inc., Cary, NC) and R (version 4.1.1, https://www.r-project.org). All patients receiving treatment were included in the full analysis set (FAS). All outcomes were analyzed in the intention-to-treat (ITT) population.

## 3 Discussion

**FIGURE 1:**
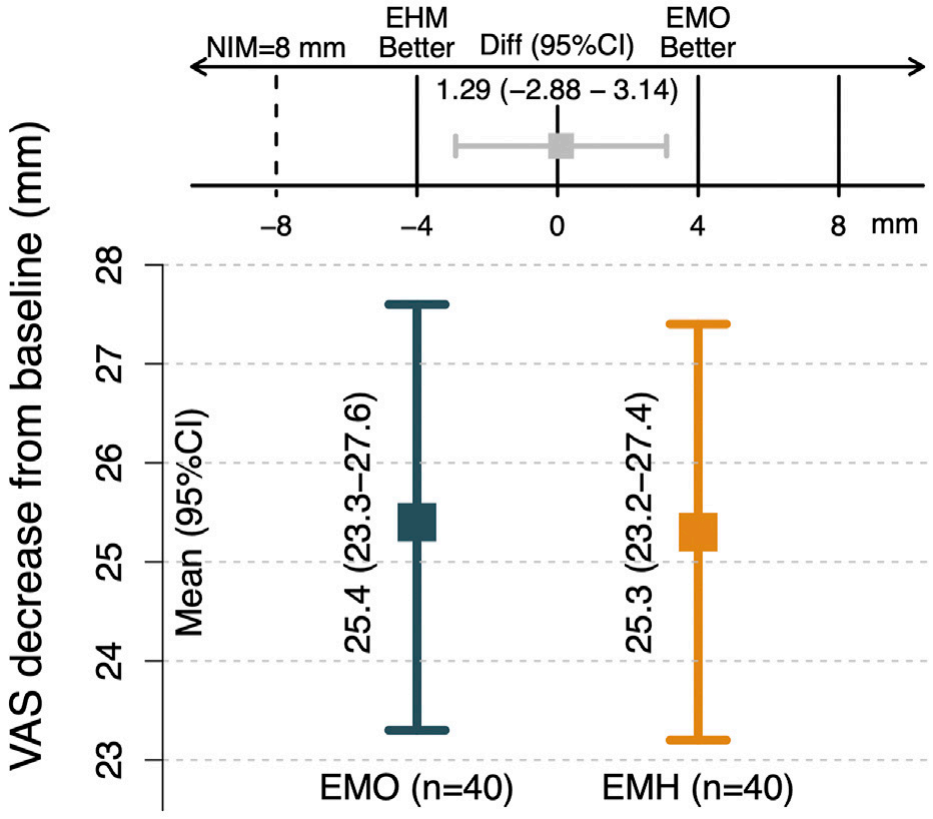
Comparison of non-inferiority of VAS between two groups The VAS decrease relative to baseline after one week of treatment was the primary outcome; 25.4- and 25.3-mm decrease from baseline of the patients in the EMO and EMH group, respectively. The 95% CI for the intergroup difference was 2.88 to 3.14. According to the 8 mm noninferiority criteria, EMH group was non-inferior to EMO in terms of the VAS decrease relative to baseline after one week of treatment.

**FIGURE 2:**
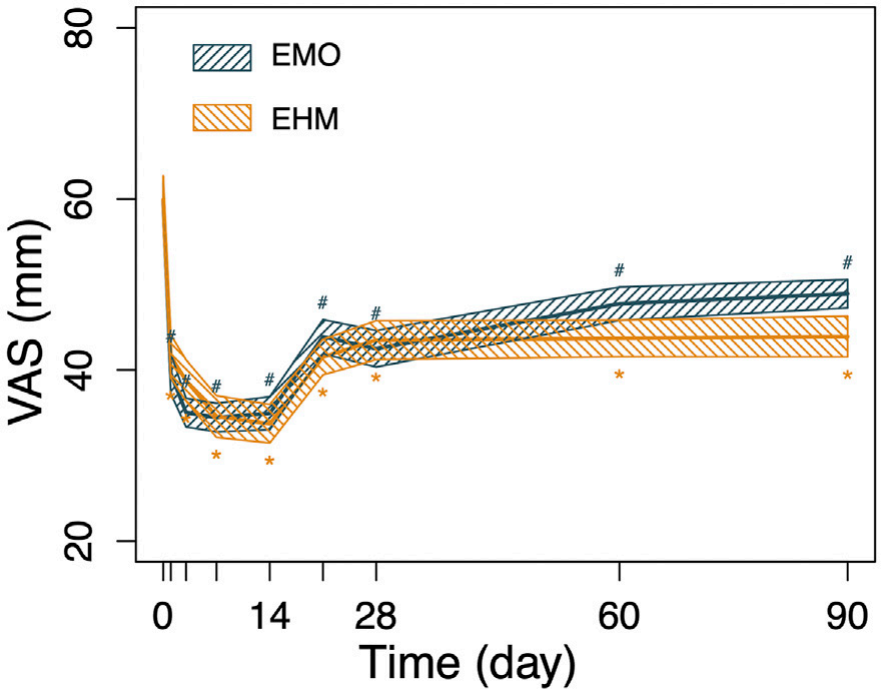
The changes in VAS of the 2 groups before and after treatment. The VAS of the two groups on day 1, day 3, day 7, and day 14 after treatment was significantly lower than before treatment. The EMO group decreased from 59.8 to 39.6 (day 1 after treatment), 35 (day 3 after treatment), 34.5 (day 7 after treatment) days) and 34.2 (day 14 after treatment), the EHM group decreased from 59.9 to 41.6 (day 1 after treatment), 38.8 (day 3 after treatment), 34.6 (day 7 after treatment) and 33.7 (day 14 after treatment). There were significant differences between the two groups compared with pretreatment (*p* < 0.05), On day 21, day 28, day 60, and day 90 after treatment, the VAS of the two groups gradually increased, but it was still lower than before treatment (*p* < 0.05). The comparison of changes in VAS relative to baseline between the two groups at different time points showed that the EMH group was better than the EMO group at 90 days (*p* < 0.05), and there was no significant difference at other time points (*p* > 0.05). Using the repeated measures analysis of variance, no significant difference was observed for the decrease of VAS relative to baseline within the two groups (*p* = 0·680); however, there was a significant difference for time and time-group interactions (*p* < 0.001, *p* < 0.001) which indicating the VAS significant decreased from baseline in the two groups and the decreased amplitude with time across the two treatment groups were significantly different.

The results of this single-center, prospective, double-blinded, randomized controlled, and non-inferiority clinical study showed that EHM was non-inferior to EMO in terms of the VDRB after 1-week treatment for intractable postherpetic neuralgia. The results in Table 1, Figures 1, 2 of this study show that even after standard treatment according to neuropathic pain guidelines, there are still patients with refractory PHN who not only have moderate or high pain scores but also exhibit marked anxiety and depression. Through the application of epidural morphine (EMO) or epidural hydromorphone (EHM), pain was significantly improved on the first day of treatment accompanied by a significant reduction in the number of breakthrough pain lasting until 14 days after treatment cessation. At the same time, effective analgesia brings about improvements in patients’ QOL, GAD-7, and PHQ-9 levels. The benefits of this clinical treatment can be discussed from the following two aspects: 1. Can opioids be used in the treatment of neuropathic pain? How is the effect? 2. If available, how can opioids be used to maximize the clinical benefit to PHN patients?

**FIGURE 3:**
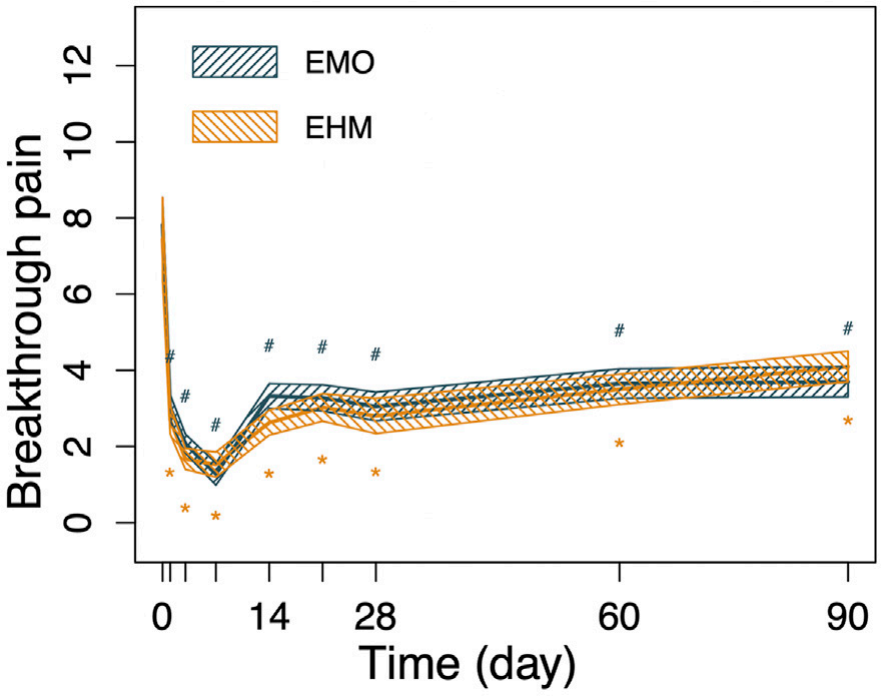
The changes in BTP of the 2 groups before and after treatment. Breakthrough pain shows similar trends to VAS, Breakthrough pain was significantly reduced in both groups after treatment, and dropped to the lowest level on the 7th day after treatment. Breakthrough pain was 1.3 per day in the EMO group, compared with 1.5 per day in the EHM group. From the 14th day after the treatment to the 90th day after the treatment, although the number of burst pains has recovered. There are still significant lower than baseline (*p* < 0.05). There was no significant difference between the two groups in breakthrough pain before and after treatment (*p* > 0.05) at each visit time. Using the repeated measures analysis of variance, no significant difference was observed for the decrease of BTP from baseline within the two groups (*p* = 0·764); however, there was a significant difference for time and time-group interactions (*p* < 0.001, *p* < 0.049) which indicating the BTP significant decreased from baseline in the two groups and the decreased amplitude with time across the two treatment groups were significantly different.

We will start with the first question. Opioids have long been considered of limited value in the treatment of neuropathic pain. However, in fact, opioids have been used for pain relief for more than a thousand years. The concept of neuropathic pain is only a few decades old. It would seem that there must have been a very long history of opioid therapy for NP before NP was even defined (Katz and Benoit, 2005). Similarly, research also shows that opioids have a definite effect on pain relief (Dellemijn and Vanneste, 1997; Foley, 2003; Watson et al., 2004). Numerous studies suggest that opioids can be effective in treating typical NP, ranging from PHN and DPN to peripheral neuralgia and phantom limb pain. The opioids discussed here refer to different types of preparations such as morphine, oxycodone, and methadone, which are not limited to one specific opioid (Watson and Babul, 1998; Maier et al., 2002; Raja et al., 2002; Morley et al., 2003). In addition, the clinical treatment effect was reconfirmed in the four studies mentioned previously. Combining the short-term and long-term effects of this study (Figures 2, 3), it shows that opioids can improve pain scores in patients with refractory PHN, which is ineffective in other treatments. Opioids can be an important part of the neuropathic pain treatment arsenal (Katz and Benoit, 2005; Cooper et al., 2017; Centers for Disease Control and Prevention, 2018). However, the standard and rational use of opioids is the most important clinical consideration. Optimizing opioid therapy requires balancing efficacy with the prevention and treatment of side effects including endocrine complications (Abs et al., 2000).

**FIGURE 4:**
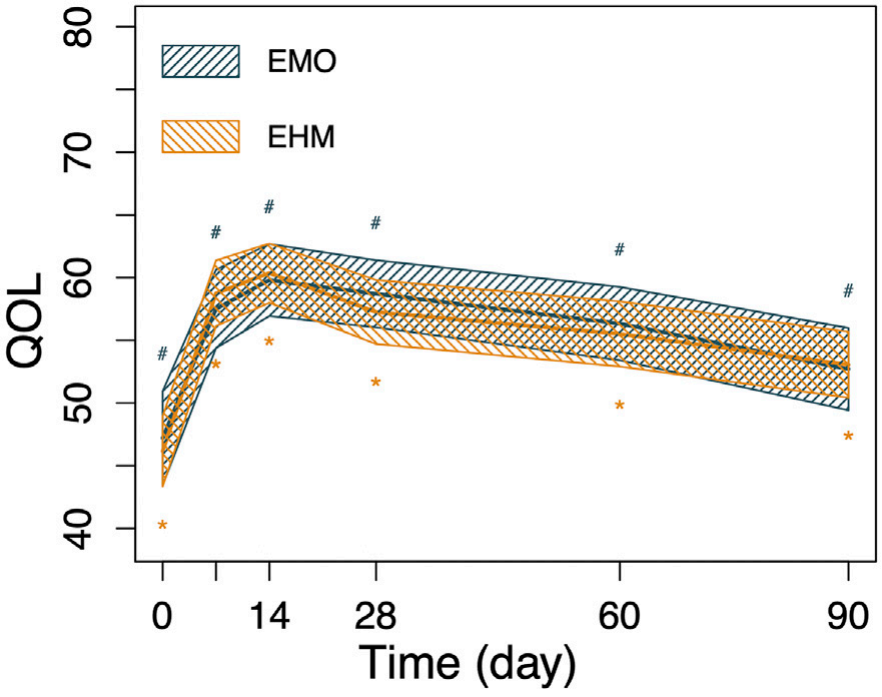
The changes in QOL of the 2 groups before and after treatment. Nonparametric Wilcoxon signed-rank test was applied to the analysis of QOL. The QOL of both groups was significantly improved on the 7th day and 14th day after treatment (*p* < 0.05), at day 14 after treatment, the QOL in the EMO group improved from 47.2 to 59.8 (*p* < 0.05), QOL in the EHM group improved from 46.2 to 60.4, QOL decreased slowly in both groups from the 14th day to 90th day, however, there was still a significant improvement compared with pretreatment (*p* < 0.05). The observed in QOL between the two groups before and after treatment were different on Day 7 (*p* <0.05). No significant differences are observed at other times (*p*> 0.05).

**FIGURE 5:**
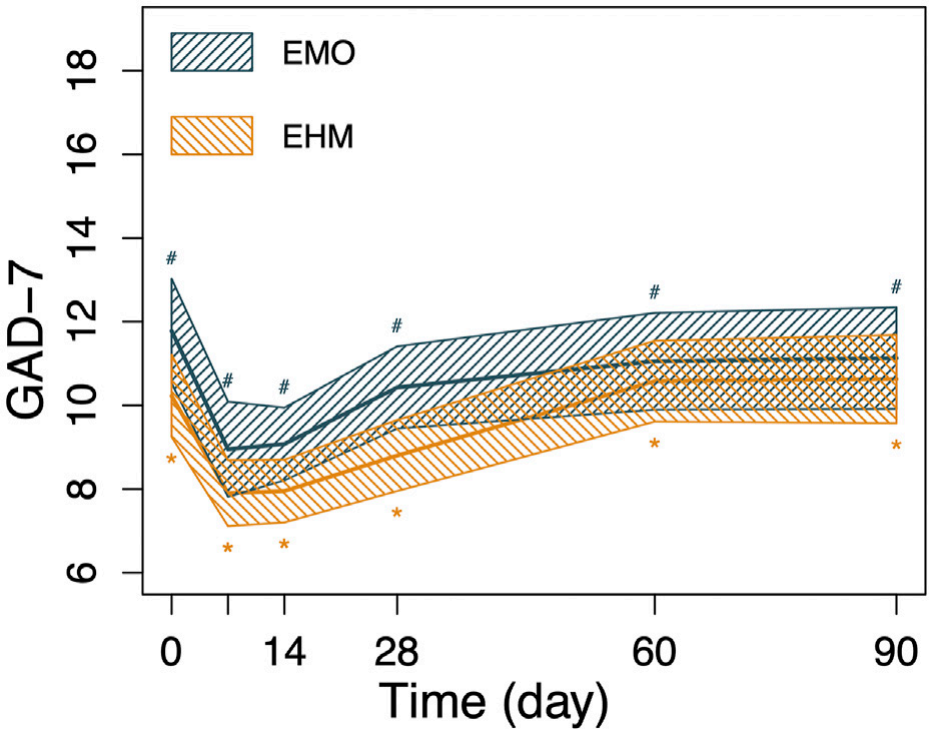
The changes in GAD-7 and PHQ-9 of the 2 groups before and after treatment. Nonparametric Wilcoxon signed-rank test was applied to the analysis of GAD-7 and PHQ-9. GAD-7 and PHQ-9 in both groups decreased significantly on the 7th day after treatment compared with before treatment (*p* < 0.05), PHQ-9 and GAD-7 decreased to 12.1 and 9.0 on the 7th day after treatment, respectively in the EMO group and 11.6 and 7.9 in the EHM group, respectively. The two scores slowly rebounded after the 14th day. However, there is still a significant higher than before treatment (*p* < 0.05). There were no significant differences in PHQ-9 and GAD-7 between the two groups at any visit time (*p* > 0.05).

**FIGURE 6:**
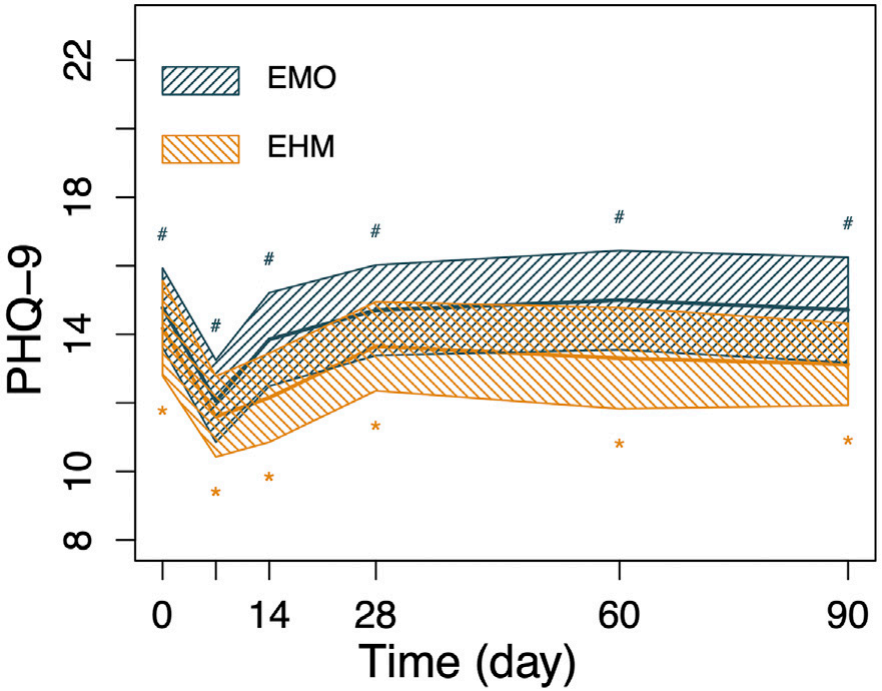
The changes in GAD-7 and PHQ-9 of the 2 groups before and after treatment. Nonparametric Wilcoxon signed-rank test was applied to the analysis of GAD-7 and PHQ-9. GAD-7 and PHQ-9 in both groups decreased significantly on the 7th day after treatment compared with before treatment (*p* < 0.05), PHQ-9 and GAD-7 decreased to 12.1 and 9.0 on the 7th day after treatment, respectively in the EMO group and 11.6 and 7.9 in the EHM group, respectively. The two scores slowly rebounded after the 14th day. However, there is still a significant higher than before treatment (*p* < 0.05). There were no significant differences in PHQ-9 and GAD-7 between the two groups at any visit time (*p* > 0.05).

The second question is that since opioids are useful, what clinical treatment strategies bring more benefits to patients? What kind of a treatment strategy suits the treatment of PHN including factors such as the route of administration, the time of administration, or the type of drug? On this issue, there is no specific guidance on which type of opioid is better indicated than the other (Manchikanti et al., 2017; Balzani et al., 2021). The results of previous studies and the results of our study (Figures 2–6) show that the use of short-term epidural opioids (morphine and hydromorphone) can provide reliable pain relief when PHN treatment is not effective. From a pharmacological point of view, the dose ratio of epidural and oral administration is 1:30 (Deer et al., 2017). Epidural opioid administration provides as much clinical analgesia in much smaller doses. Meanwhile, the continuous infusion method in this study ensured a stable drug concentration. This method of administration brought a definitive analgesic effect starting on day 1 of the treatment, and good pain relief lasted even 14 days after the treatment cessation. Satisfactory pain relief improved the patient’s quality of life, decreased their anxiety/depression level, and the patient’s overall quality of life benefited from the effective analgesia. Importantly, no significant side effects such as severe nausea, vomiting, or constipation were found in the EMO or EHM groups. There were no concerning complications such as puncture-related infection which indicates a good safety profile. Considering the onset time of effective analgesia, drug dose, analgesic efficacy, and other aspects, short-term use of both EMO and EHM brings relatively rapid and safe analgesic effects on refractory PHN patients. However, if long-term use is required, more drug-related side effects should be noted (Hoffman et al., 2017; Sommer, 2017)^.^

The results in Figure 8 suggest that there is a positive trend between the decrease of VAS and the improvement of QOL and the improvement of GAD-7 and PHQ-9 in this study. This presents a reasonable basis for the theory of “comorbidity mechanisms” regarding chronic pain and anxiety–depressive states. It is well known that the comorbidity of chronic pain and psychiatric disorders (such as depression and anxiety) has become a clinical consensus. The underlying mechanism is mainly associated with central sensitization (Li et al., 2020; Tang et al., 2021). Among them, anxiety and depression caused by PHN are particularly common in clinical practice. Since the PHN virus directly damages DRG neurons, which leads to central sensitization, PHN often shares the same central brain nuclei with anxiety/depression disorders (Li et al., 2018; Du et al., 2021). The pain relief provided by epidural opioids may reduce the negative emotional, cognitive, and behavioral effects of intractable pain and ultimately improve the patient’s quality of life.

Looking into the treatment inclusion criteria and treatment results of this study, it appears that the clinical benefit of opioid use requires attention to the following issues: the first thing to note is that one of the foundations for a good clinical treatment effect lies in a comprehensive clinical evaluation to ensure a correct and comprehensive diagnosis.

The second point to note is that opioids should only be considered if first-line drugs (tricyclic antidepressants, serotonin–norepinephrine reuptake inhibitors (SNRIs), and gabapentin) alone or in combination cannot manage to control pain satisfactorily. Therefore, it is important to ensure adherence to treatment regimens using these drugs before considering opioids (Javed et al., 2015; Balhara et al., 2020).

**FIGURE 7:**
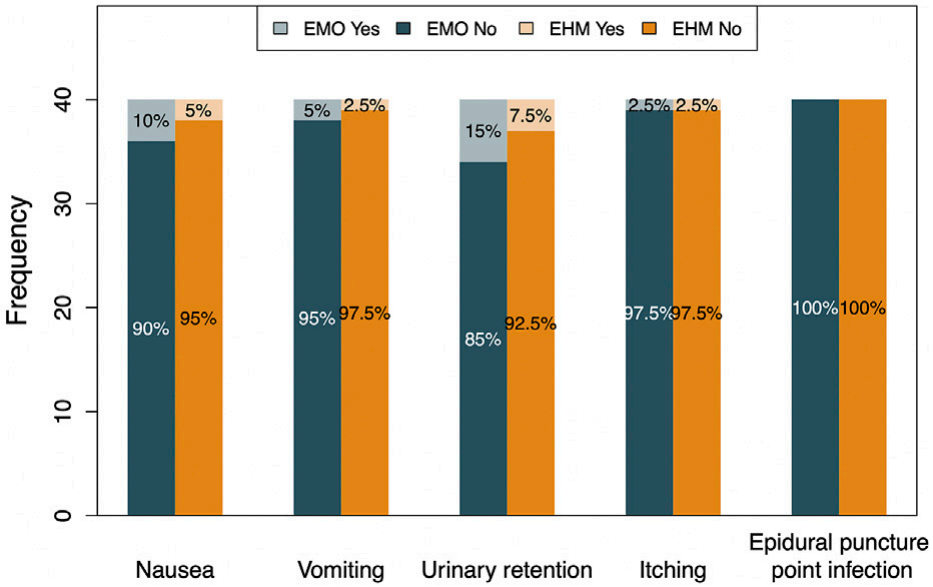
The incidence of adverse reactions between the two groups The most common adverse reactions after treatment are nausea, vomiting, urinary retention. The incident rate of nausea, vomiting, and urinary retention, are 10% (4/40), 5% (2/40), and 15% (6/40), respectively, in EMO group,. While, 5% (2/40), 2.5% (1/40) and 7.5% (3/40), respectively, in EHM group. The probability of AE in EMO group was higher than that of EHM group (*p* > 0.05), but considering low incidence of AE, there was not statistically meaning. The overall probability of itching was lower than the other three adverse reactions, 2.5% (1/40) incidence observed in both groups (*p* > 0.05). The above-mentioned adverse reactions were all relatively mild, except for 1 patient in the EMO group and 1 patient in the EHM group who received catheterization. There was no infection at the epidural puncture site in both groups. At the end of the trial, we found that almost no OWS was reported between the two groups. The EMO group only reported 1 case of palpitations and 1 case of sweating on the 4th day after treatment; the EHM group reported 1 case of sweating on the 1st day after treatment, 2 cases of yawning on the 4th day after treatment. The limited AE events, less two days AE duration and mild symptoms indicates that no need to special treatment.

Undoubtedly, in the clinical use of opioids, in addition to clinical efficacy, how to avoid the adverse effects of opioids and potential withdrawal reactions is a very important safety issue. Figure 7 shows that the side effects of EMO and EHM are mainly nausea, vomiting, urinary retention, etc. The overall incidence is low, and the symptom is relatively mild. Generally, no special treatment is required, and most of them disappear over time. This reflects the clinical advantages of short-term, low-dose opioid use in this study, avoiding the lasting side effects of long-term, high-dose opioid use. Through the comparison of side effects, it can be seen that the EHM group shows more clinical advantages, which is consistent with the pharmacologic profile of hydromorphone with a suitable partition coefficient and its better analgesic efficacy (Ma et al., 2020). At the same time, it is also necessary to pay attention to the combined use of drugs when observing the side effects of drugs. The combination of drugs increases the incidence of side effects. Opioids combined with gabapentin, although more effective in treating NP, are also associated with a higher risk of adverse drug reactions than taking these drugs alone. Although there were no higher side effects in the two treatment groups in this study, EHM had a slight advantageous trend over EMO in terms of its safety profile. It deserves a more detailed and timely observation of side effects.

**FIGURE 8:**
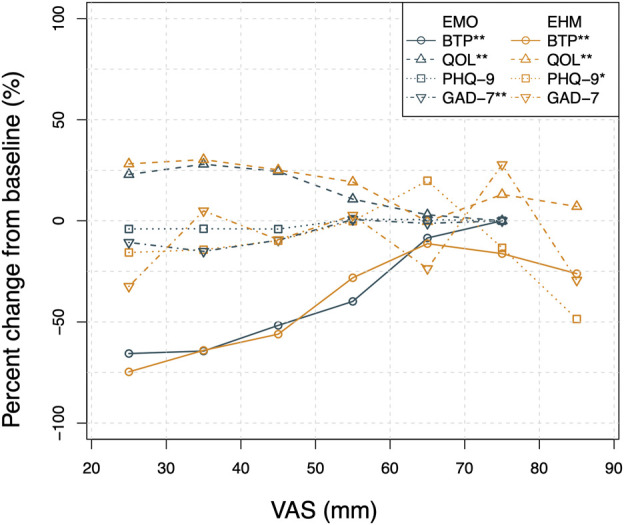
The correlation plot of BFT\QOL\ PHQ-9\ GAD-7 versus VAS by treatment group. The Pearson correlation analysis of BFT, QOL, PHQ-9, and GAD-7 change from baseline versus VAS show a low level of correlation (*r* < 0.5) mainly due to the variability and limited sample size. However,as shown in the Figure 8, the correlation plot by treatment group clearly shows that the average BFT percent change from baseline decreases rapidly with the VAS decrease from 80 to 40 mm while a relatively flat trend is observed below 40 mm for both groups. The improvement of the average change of QOL was apparently associated with the decrease in VAS. The average change of PHQ-9, and GAD-7 scores show a similar positive relationship with VAS. Linear regressions were applied to all the relationships. The estimated p-values of slope of linear regression are smaller than 0.05 except PHQ-9 of EMO group and GAD-7 in EHM group.

Only a few opioid withdrawal symptoms including palpitations, lacrimation, sweating, and yawning were observed in both groups after the cessation of epidural opioids, as shown in the Supplementary Data (Overall data. xlsx). Generally speaking, OWS begins soon after opioid discontinuation, are often severe, and may motivate patients to restart opioids in the early days after opioid discontinuation or prevent them from attempting to stop opioids at all (Kosten and Baxter, 2019). Early diagnosis of OWS and symptomatic treatment are very important. The occurrence of OWS is closely related to factors such as the half-life of the opioid used, the duration of opioid use, and the specific characteristics of each patient (including health status). No obvious OWS was observed in this study, which may be related to factors such as short-term (3-day) use, low-dose morphine and hydromorphone, and stable plasma concentrations under continuous application. The continuous epidural application provides stable blood drug concentrations, and no significant fluctuations between drug peaks and troughs play an important role in reducing the occurrence of OWS.

### 3.1 Limitations to this study

In the demographic background information collection of this study, data on opioid use for analgesia could not be successfully collected, which is a weakness in the study, because the patients’ recollection about this issue was vague, and hence the background information was not collected completely. Since this clinical study was not intended to compare the pros and cons of oral and epidural opioid application, hopefully a future multicenter study will further emphasize and optimize the collection of relevant data. In the establishment of observation variables, the score of neuropathic pain such as ID pain or DN4 was not used as one of the indicators for evaluation, which is also one of the limitations. As a single-center study, the number of cases is not large enough. Further research on this topic should be conducted through multicenter studies to optimize the use of opioids in NP.

## 4 Conclusion

In summary, EHM was non-inferior to EMO in terms of the VDRB after 1-week treatment. Epidural opioids (EMO and EHM) can provide rapid pain relief, improve quality of life, and improve related anxiety/depression states in PHN patients whose VAS remains greater than 50 mm despite first-line therapy according to IASP and CASP. Patients receiving epidural opioids had no obvious side effects or OWS after drug withdrawal. EMO and EHM can be considered one clinical option for PHN patients who are resistant to conservative treatment.

## Data Availability

The raw data supporting the conclusion of this article will be made available by the authors, without undue reservation.
